# Endoscopic Ultrasound-Guided Hepaticogastrostomy for a Benign Biliary Stricture in a Patient Allergic to Both Iodinated and Gadolinium Contrast Media: A Case Report

**DOI:** 10.7759/cureus.77072

**Published:** 2025-01-07

**Authors:** Koji Takahashi, Hiroshi Ohyama, Izumi Ohno, Naoya Kato

**Affiliations:** 1 Gastroenterology, Chiba University, Chiba, JPN

**Keywords:** allergic, endoscopic retrograde cholangiopancreatography (ercp), endoscopic ultrasound-guided hepaticogastrostomy, gadolinium contrast, iodinated contrast media

## Abstract

Allergic reactions to iodinated and gadolinium contrast media, though rare, present significant challenges in managing biliary obstructions. This case report describes a novel approach to biliary drainage in a patient with such allergies using endoscopic ultrasound-guided hepaticogastrostomy (EUS-HGS). An 81-year-old woman with multiple allergies, including to both iodinated and gadolinium contrast media, developed a benign biliary stricture following repeated radiofrequency ablation for hepatocellular carcinoma. Standard endoscopic retrograde cholangiopancreatography was contraindicated due to these allergies. An EUS-HGS was successfully performed, achieving effective biliary drainage through ultrasound-guided puncture and stent placement without the use of contrast agents. The procedure was complication-free, and the patient’s hepatobiliary enzyme levels improved significantly postoperatively. An EUS-HGS offers a safe and effective alternative for biliary drainage in patients with dual contrast agent allergies, especially when conventional methods are not viable. This case highlights the potential of EUS-HGS in addressing complex clinical challenges.

## Introduction

Biliary drainage is crucial for managing cholestasis, with endoscopic methods like endoscopic nasobiliary drainage (ENBD) or stent placement being preferred due to lower morbidity and shorter hospital stays [1]. Endoscopic retrograde cholangiopancreatography (ERCP) typically relies on iodinated contrast media; however, in rare cases, patients may exhibit allergies to iodinated contrast media. Gadolinium contrast media is an alternative to iodinated contrast media, although it has inferior fluoroscopic visibility compared to iodinated contrast media. However, there are very few patients who are allergic to both [2].

This case report describes a patient who experienced a benign bile duct stricture after undergoing repeated radiofrequency ablation (RFA) for hepatocellular carcinoma (HCC) and presented with dual contrast media allergies. Endoscopic ultrasound-guided hepaticogastrostomy (EUS-HGS) was utilized as a reliable and effective alternative for biliary drainage. An EUS-HGS enables precise visualization and bile duct puncture using ultrasound guidance, allowing for the creation of an internal fistula in a single session. This report highlights the value of EUS-HGS in addressing complex medical challenges.

## Case presentation

**Figure 1:**
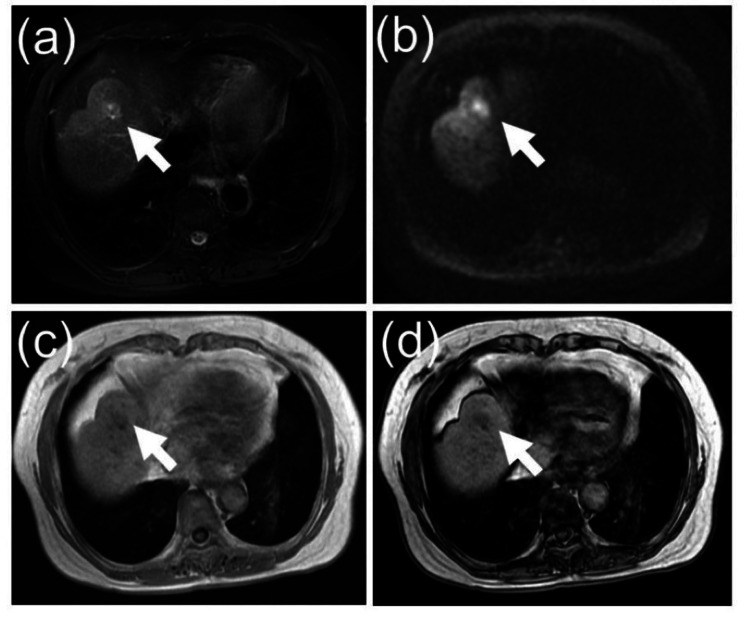
Non-contrast MRI images of recurrent hepatocellular carcinoma There is a 17 mm hepatocellular carcinoma (white arrow) in segment IV of the liver, which shows high signal intensity on fat-suppressed T2-weighted images and diffusion-weighted images, with the signal decreasing from in-phase to out-of-phase. (a) Fat-sat T2-weighted image; (b) Diffusion-weighted image; (c) T1-weighted in-phase image; (d) T1-weighted out-of-phase image.

An 81-year-old woman with a history of anaphylactic reactions to various allergens, including iodinated and gadolinium contrast media, salmon, and squid, was referred to our institution for evaluation and management of cholestasis caused by a biliary stricture in the left hepatic duct. Her medical history included hepatitis C virus-related cirrhosis (genotype 1b), for which she achieved a sustained virologic response eight years ago following treatment with ledipasvir/sofosbuvir. She had undergone RFA seven years ago for a 15-mm HCC in segment IV of the liver, with additional RFA sessions performed two years later. Most recently, she underwent RFA four months ago for a 17-mm recurrent HCC in the same liver segment (Figure 1).

**Figure 2:**
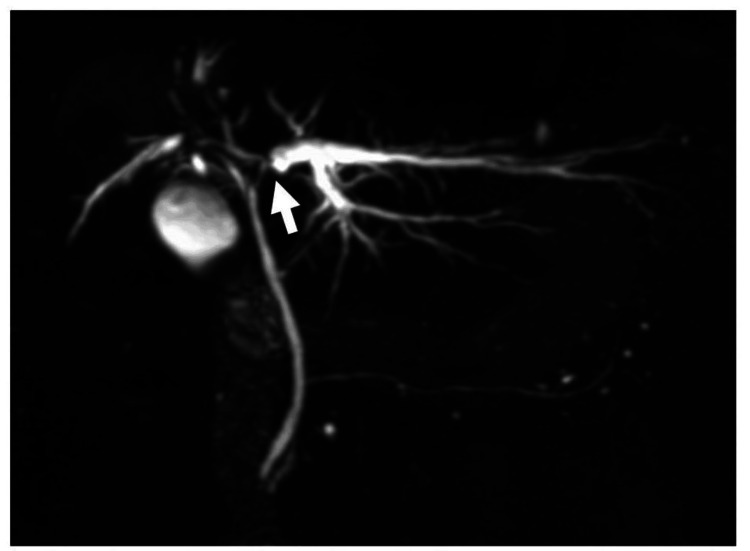
Magnetic resonance cholangiopancreatography image reveals a stricture (white arrow) in the left hepatic duct, accompanied by dilation of the peripheral bile ducts (B2 and B3).

Following the most recent RFA, blood tests revealed elevated hepatobiliary enzyme levels. Magnetic resonance cholangiopancreatography showed a stricture in the left hepatic duct with peripheral ductal dilation (Figure 2).

Conventional ERCP was contraindicated due to her dual contrast media allergies. Given her cholestasis, biliary drainage was deemed necessary.

She was subsequently referred to our hospital for biliary drainage. When she visited our hospital, her blood tests showed elevated hepatobiliary enzyme levels without jaundice (Table 1).

**Table 1:** Laboratory data WBC: white blood cell; RBC: red blood cell; Hb: hemoglobin; Hct: hematocrit; MCV: mean corpuscular volume; MCH: mean corpuscular hemoglobin; MCHC: mean corpuscular hemoglobin concentration; Plt: Platelet; TP: total protein; Alb: albumin; BUN: blood urea nitrogen; Cre: creatinine; Na: sodium; K: potassium; Ca: calcium; AST: aspartate aminotransferase; ALT: alanine transaminase; LDH: lactate dehydrogenase; ALP: alkaline phosphatase; γ-GTP: gamma-glutamyl transpeptidase T.Bil: total bilirubin; D.Bil: direct bilirubin; Amy: amylase; CK: creatine kinase; CRP: C-reactive protein

Parameter	Value	Reference range
WBC	4,700 /μL	3,500-9,700
RBC	453×10^4 ^/μL	376-516
Hb	13.6 g/dL	11.2-15.2
Hct	42.2 %	34.3-45.2
MCV	93.2 fL	80-101
MCH	30.0 pg	26.4-34.3
MCHC	32.2 %	31.3-36.1
Plt	16.5 ×10^4 ^/μL	14.0-37.9
TP	7.5 g/dL	6.5-8.2
Alb	4.7 g/dL	3.8-5.2
BUN	15.0 mg/dL	8-20
Cre	0.70 mg/dL	0.46-0.82
Na	140 mEq/L	135-145
K	4.2 mEq/L	3.5-5.0
Ca	9.9 mg/dL	8.6-10.2
AST	101 IU/L	10-40
ALT	110 IU/L	5-45
LDH	240 IU/L	120-245
ALP-IF	159 IU/L	38-113
γ-GTP	1150 IU/L	0-48
T.Bil	1.0 mg/dL	0.3-1.2
D.Bil	0.2 mg/dL	0-0.4
Amy	94 mg/dL	39-134
CK	46 IU/L	50-210
CRP	0.11 mg/dL	0.00-0.30

No tumor was identified near the biliary stricture, suggesting a benign etiology secondary to previous RFA. Endoscopic biliary stenting was prioritized for drainage. However, ERCP was deemed unsuitable for the following reasons: 1) In the absence of contrast media, it is challenging to confirm whether the guidewire has entered the pancreatic duct or bile duct, even if initially inserted; 2) If the guidewire inadvertently enters the pancreatic duct, advancing it further risks perforation; 3) Even if the guidewire successfully reaches the bile duct, it may fail to traverse the stenotic segment; and 4) There is a risk of stent placement in an unintended location. Given these considerations, EUS-HGS was chosen as it enables guidewire insertion by puncturing the bile duct under direct ultrasound guidance, specifically targeting areas of bile stasis.

**Figure 3:**
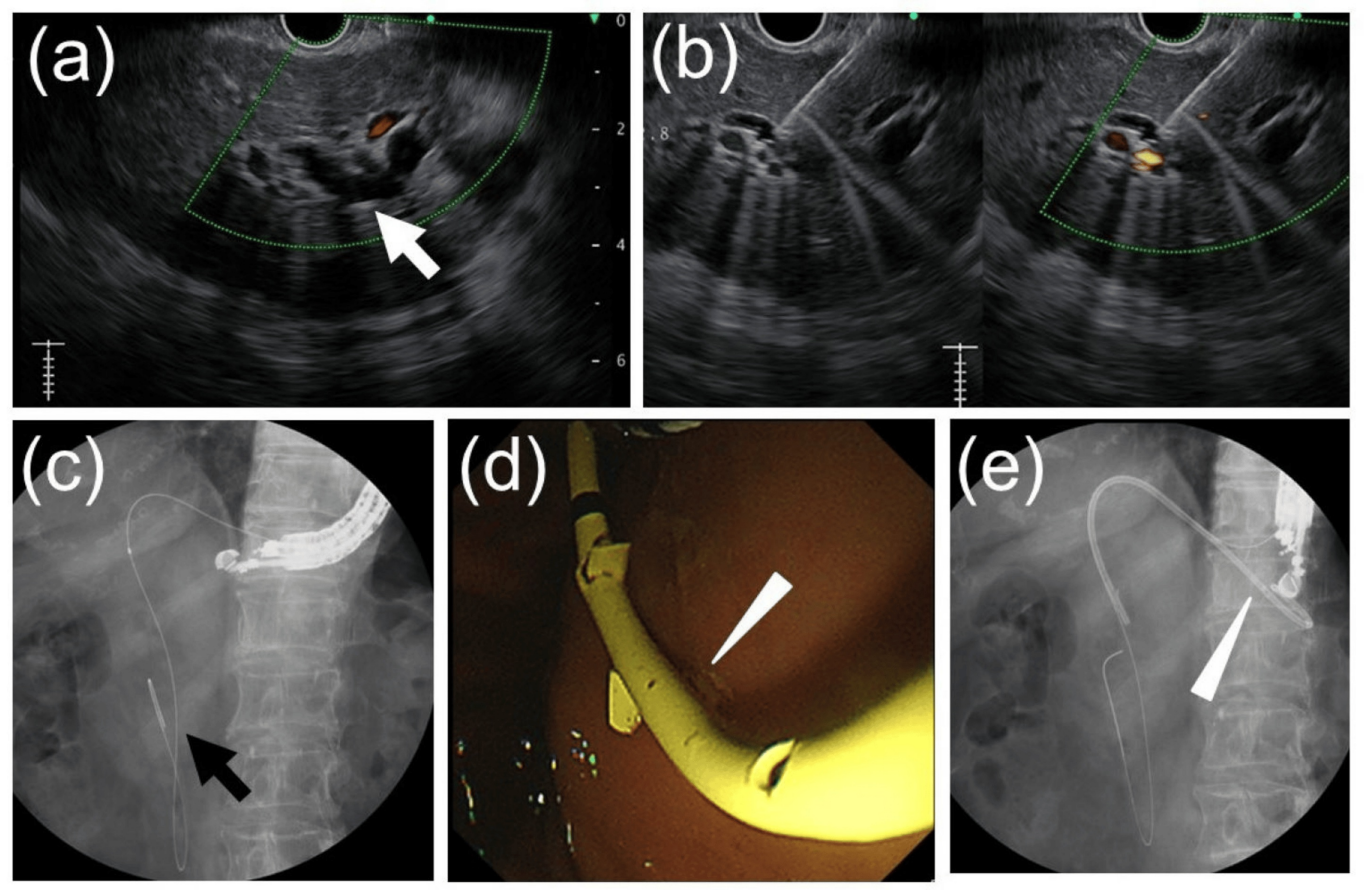
Images obtained during endoscopic ultrasound-guided hepaticogastrostomy (a) The dilated left intrahepatic bile duct (white arrow) was visualized using an ultrasound endoscope from the stomach. (b) The dilated left intrahepatic bile duct was punctured under endoscopic ultrasound guidance. (c) A guidewire (black arrow) was inserted into the bile duct and advanced beyond the duodenal papilla into the duodenal lumen. (d) An additional guidewire was advanced into the bile duct, and a plastic stent (white arrowhead) was inserted transgastrically into the bile duct, with one guidewire left in place. (e) A plastic stent (white arrowhead) was deployed from the stomach into the bile duct, successfully completing endoscopic ultrasound-guided hepaticogastrostomy.

An EUS-HGS was performed using a GF-UCT260 echoendoscope (Olympus, Tokyo, Japan). The B3 intrahepatic bile duct was punctured from the stomach with a 19-gauge EZ Shot 3 Plus puncture needle (Olympus, Tokyo, Japan). A VisiGlide 2 guidewire (Olympus, Tokyo, Japan) was advanced through the biliary stricture into the duodenal lumen. The puncture site was dilated using a 4-mm REN balloon catheter (Kaneka Medix, Osaka, Japan), and bile was aspirated to confirm proper guidewire placement within the bile duct. As the guidewire passed through the biliary stricture and extended toward the extrahepatic bile duct, it was determined that placing a stent along this guidewire would result in its tip being located in the extrahepatic bile duct, which would inadequately drain the area of cholestasis. To address this, we decided to place two biliary stents. A second guidewire, Endoselector (Boston Scientific, Marlborough, MA, USA), was inserted through the catheter. Subsequently, the first biliary plastic stent, a 7Fr, 14-cm Through & Pass TYPE IT stent (Gadelius Medical, Tokyo, Japan), was placed with its tip positioned in the extrahepatic bile duct (Figure 3).

**Figure 4:**
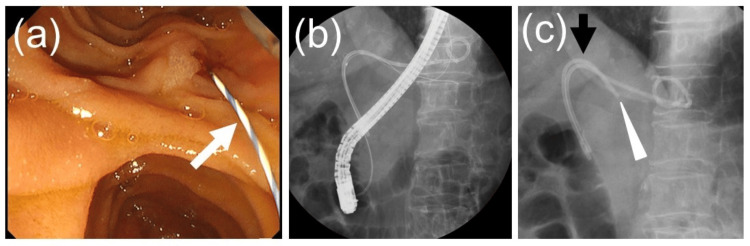
Images of transpapillary inside stent placement performed using the rendezvous method after ultrasound-guided hepaticogastrostomy. (a) The scope was advanced to the descending part of the duodenum, and the guidewire protruding from the duodenal papilla was confirmed. (b) The bile duct was accessed using a rendezvous technique using a guidewire. (c) An inside stent was deployed transpapillary to cross the stricture in the bile duct, completing the procedure.

The second stent was deployed transpapillary using the rendezvous technique as follows: the endoscope was exchanged for a TJF-Q290V (Olympus, Tokyo, Japan) and advanced to the descending duodenum. Utilizing the guidewire extending from the duodenal papilla into the duodenal lumen, a 7Fr, 12-cm Through & Pass-inside deep-angle stent was positioned above the papilla, successfully crossing the biliary stricture (Figure 4).

The procedure was completed without complications. Postoperatively, the patient’s hepatobiliary enzyme levels showed significant improvement. She was scheduled for regular HCC surveillance and potential stent replacement as needed in case of dysfunction.

## Discussion

This case highlights the effectiveness of EUS-HGS as an alternative biliary drainage method for patients allergic to both iodinated and gadolinium contrast media. The procedure bypassed the limitations of standard ERCP by utilizing ultrasound guidance without requiring contrast agents. The success of this approach underscores the importance of innovative techniques for managing complex clinical scenarios where conventional methods are contraindicated.

The rarity of dual contrast agent allergies cannot be overstated. Allergic reactions to iodinated contrast media occur in approximately 0.05%-0.1% of radiological procedures, with severe reactions occurring even less frequently at 0.02%-0.5% [3-5]. Similarly, gadolinium contrast media is associated with a hypersensitivity reaction incidence of 9.2 per 10,000 administrations, with severe reactions reported in 0.52 per 10,000 cases [6]. Allergies to both agents are exceedingly rare, with an incidence of 0.047% among patients exposed to both [7]. These statistics highlight the need for alternative strategies in such exceptional cases.

Benign biliary strictures are recognized as a complication of repeated RFA for HCC, with reported incidence rates ranging from 1.8% to 12% [8,9]. Factors contributing to these strictures include repeated ablations, tumor proximity to bile ducts, and vascular involvement. Proactive measures, such as cooling the bile duct by infusing chilled saline solution via an ENBD tube during RFA, have been suggested to reduce this risk [10]. However, this method is not widely adopted, highlighting the importance of vigilant follow-up and timely intervention.

Endoscopic ultrasound-guided hepaticogastrostomy has gained prominence as a minimally invasive and highly effective technique for biliary drainage, with reported technical success rates of 89%-100% and clinical success rates of 88.1%-100% in both malignant and benign biliary obstructions [11-13]. Despite its high success rates, complications such as cholangitis, bleeding, and stent migration remain potential risks, with rates ranging from 6.9% to 41.7% [11,12]. In the present case, the successful placement of two stents, one via EUS-HGS and the other using a rendezvous ERCP technique, demonstrates the flexibility of this approach in addressing complex biliary strictures.

Alternative techniques, such as air cholangiogram and carbon dioxide cholangiogram, have been reported [14,15]. However, these techniques require safe and precise bile duct access without contrast, which can pose significant challenges. In this case, EUS-HGS provided a more reliable option for bile duct access and drainage, particularly given the patient’s multiple allergies and the potential risks associated with ERCP.

The implications of this case extend beyond the immediate clinical outcome. It underscores the importance of tailoring interventions to individual patient needs, particularly in scenarios where standard techniques are contraindicated. The use of EUS-HGS without contrast agents is a novel approach that may inspire further studies aimed at refining this technique and broadening its applications. Furthermore, it highlights the necessity for interdisciplinary collaboration in managing patients with unique challenges, such as multiple allergies. The successful outcome in this case highlights the potential of EUS-HGS as a primary treatment option in comparable clinical contexts. Future research should focus on long-term outcomes, procedural refinements, and the development of specialized stents to further improve safety and efficacy. As operator experience with EUS-HGS grows, its application in managing complex biliary obstructions is likely to expand, benefiting a wider range of patients.

## Conclusions

Patients with dual allergies to iodinated and gadolinium contrast media pose distinct challenges in managing biliary obstructions. This case demonstrates that EUS-HGS is a safe and effective alternative for biliary drainage, providing a practical solution when standard ERCP is not viable. The procedure was completed successfully without complications, resulting in a marked improvement in the patient’s hepatobiliary enzyme levels. Long-term care included surveillance for hepatocellular carcinoma and close monitoring for potential stent dysfunction. This case highlights the need for individualized approaches to complex cases and supports the potential of EUS-HGS as a primary treatment option in similar situations.
